# Supplementary material for: Long COVID manifests with T cell dysregulation, inflammation and an uncoordinated adaptive immune response to SARS-CoV-2
Source: Nat Immunol. 2024 Jan 11;25(2):218–25. doi: 10.1038/s41590-023-01724-6 (PMC10834368; doi:10.1038/s41590-023-01724-6)
Supplement: Supplementary file 1 — Supplementary Tables 1–13. [file 41590_2023_1724_MOESM1_ESM.pdf]

nature immunology

Letter

https://doi.org/10.1038/s41590-023-01724-6

Long COVID manifests with T cell dysregulation, inflammation and an uncoordinated adaptive immune response to SARS-CoV-2

In the format provided by the authors and unedited

Supplementary Tables

Supplementary Table 1: Participants' sequelae status at the 8th-month visit

Status	N	Age[1]	M4[2]	M8[3]	Male	Female	Hos[4]	Non-Hos[5]
LC	27	46	4.5	7	10	17	7	20
R	16	45.5	0	0	9	7	2	14

Age[1]: Median age of participants involved in this study.

M4[2]: Median symptom counts at the 4th-month visit.

M8[3]: Median symptom counts at the 8th-month visit.

Hos[4]: Participants were hospitalized at the time of acute COVID-19.

Non-Hos[5]: Outpatient participants who were not hospitalized for COVID-19.

Supplementary Table 2: Detailed participants' demographics and hospitalization status at the 8th-month visit

**Participants**	**All**	**Sex**		**Hospitalized**	
		Male	Female	Yes	No
N	43	19	24	9	34
Age (Median)	46	53	43	46	48
M4[1] (Median)	2.5	0	5	7	2
M8[2] (Median)	5	2	7	10	3
Female	24	-	-	6	18
Hospitalized	9	3	6	-	-
**Race (N)**					
White	25	13	12	0	25
Latinx*	11	6	5	7	4
Black	2	2	0	1	1
Asian	3	1	2	1	2
NA#	2	2	0	0	2

M4[1]: Symptom counts at the 4th-month visit.

M8[2]: Symptom counts at the 8th-month visit.

Latinx*: Hispanic or Latino.

NA[#]: Data are not available here.

Supplementary Table 3: Participants from the LIINC cohort

Person ID	Age	Race	Status	Sex	Hospitalized	WHO Cat.[#]
102*	50	White	R	Female	No	2
111	40	White	LC	Female	No	2
147^	33	Latinx	LC	Female	No	2
161[&]	48	White	LC	Female	No	2
204	71	Asian	LC	Male	No	1
213	48	White	LC	Male	No	2
230	52	Latinx	R	Male	Yes	4-5
233	67	White	LC	Male	No	2
234	49	White	R	Female	No	1
245	71	White	LC	Female	No	2
247	66	White	LC	Male	No	2
283	19	White	LC	Female	No	2
313	48	Asian	R	Male	No	2
338	31	White	R	Female	No	2
414^	49	Latinx	LC	Female	Yes	4-5
439	68	White	R	Male	No	1
447	43	NA	LC	Female	No	2
476	65	Latinx	LC	Male	No	2
526	40	AA	R	Female	No	1
546[&]	43	AA	LC	Female	Yes	4-5
560	51	Latinx	LC	Female	No	2
564	60	White	R	Male	No	1
608*	38	NA	R	Female	No	1
621	59	White	LC	Male	No	2
652	40	White	LC	Male	No	2
670	25	Latinx	LC	Male	Yes	4-5
693	42	White	R	Male	No	2
709^	57	White	LC	Female	No	2
717	52	White	LC	Female	No	2
741	53	Latinx	R	Male	Yes	4-5
753	43	White	LC	Female	No	2
799^	46	Latinx	LC	Female	Yes	4-5
804[&]	26	White	LC	Female	No	2

831	44	Asian	LC	Female	Yes	4-5
871	34	Latinx	LC	Female	Yes	3
896	32	White	R	Male	No	2
900	31	Latinx	LC	Male	No	2
912	40	White	R	Male	No	2
913*	43	White	R	Female	No	2
914	59	White	R	Male	No	1
923	54	White	LC	Male	No	2
927[&]	46	Latinx	LC	Female	Yes	4-5
942*	37	White	R	Female	No	1

Latinx: Hispanic or Latino.

AA: Black or African American.

NA: Not available.

*Analyzed by scRNAseq (R group)

[&]Analyzed by scRNAseq (*OR7D2*^{high} LC group)

^ Analyzed by scRNAseq (*ALAS2*^{high} LC group)

[#] WHO Score determined as follows, based on the acute period of illness: 1 (ambulatory, no activity limitation) if not hospitalized and reported no problems with walking, self-care, or usual activities; 2 (ambulatory, activity limitation) if not hospitalized and any problems with walking, self-care, or usual activities; 3 (hospitalized, no oxygen therapy) if hospitalized but no supplemental oxygen administered; 4-5 (hospitalized, oxygen mask or nasal prongs or hospitalized, noninvasive mechanical ventilation [NIMV] or high flow nasal cannula [HFNC]) if hospitalized and supplemental oxygen administered. Note that this category is provided as a range because the study case report forms do not distinguish between these methods of oxygen delivery. No participant in this analysis required invasive mechanical ventilation, pressors, or extracardiac membranous oxygenation (ECMO).

Supplementary Table 4: CyTOF antibodies used in study

Antibody	Clone	Catalog	Metal	Manufacturer	Dilution
CD196/CCR6	11A9	3141014A	141Pr	Standard BioTools	1: 100
IL-4*	MP4-25D2	3142002B	142Nd	Standard BioTools	1: 200
CD38	HIT2	303535	143Nd	BioLegend	1: 100
CD195/CCR5	NP6G4	3144007A	144Nd	Standard BioTools	1: 200
CD30	BerH8	555827	145Nd	BD	1: 80

CD8a	RPAT8	3146001B	146Nd	Standard BioTools	1: 100
CXCR4	12G5	306523	147Sm	BioLegend	1: 50
CD278/ICOS	C398.4A	3148019B	148Nd	Standard BioTools	1: 200
CD25	2A3	3149010B	149Sm	Standard BioTools	1: 200
CCL4*	D211351	3150004B	150Nd	Standard BioTools	1: 100
TNF*	Mab11	3152002B	152Sm	Standard BioTools	1: 200
CD62L/L-selectin	DREG56	3153004B	153Eu	Standard BioTools	1: 200
CD95	50825	MAB326100	154Sm	R&D	1: 100
CD279/PD1	EH12.2H7	3155009B	155Gd	Standard BioTools	1: 200
CD29	TS2/16	3156007B	156Gd	Standard BioTools	1: 100
CTLA4*	14D3	5012919	157Gd	eBioscience	1: 100
CD134/OX40	ACT35	3158012B	158Gd	Standard BioTools	1: 200
CD197/CCR7	G043H7	3159003A	159Tb	Standard BioTools	1: 100
CD28	CD28.2	3160003B	160Gd	Standard BioTools	1: 100
Ki-67*	B56	3161007B	161Dy	Standard BioTools	1: 200
CD69	FN50	3162001B	162Dy	Standard BioTools	1: 200
IL-6*	MQ2-13A5	501115	163Dy	BioLegend	1: 100
CD45RO	UCHL1	3164007B	164Dy	Standard BioTools	1: 100
CD127/IL7Ra	A019D5	3165008B	165Ho	Standard BioTools	1: 200
IL-2*	MQ117H12	3166002B	166Er	Standard BioTools	1: 200
CD27	L128	3167006B	167Er	Standard BioTools	1: 200
IFNγ*	B27	3168005B	168Er	Standard BioTools	1: 200
CD45RA	HI100	3169008B	169Tm	Standard BioTools	1: 150
CD3	UCHT1	3170001B	170Er	Standard BioTools	1: 200
CD185/CXCR5	RF8B2	3171014B	171Yb	Standard BioTools	1: 100
CD57	HCD57	3172009B	172Yb	Standard BioTools	1: 200
Granzyme B*	GB11	3173006B	173Yb	Standard BioTools	1: 200
CD4	SK3	3174004B	174Yb	Standard BioTools	1: 100
Perforin*	BD48	3175004B	175Lu	Standard BioTools	1: 200
Foxp3*	206D	320102	176Yb	BioLegend	1: 100
TIGIT	MBSA43	3209013B	209Bi	Standard BioTools	1: 100
IL-17*	BL168	512331	89Y	BioLegend	1: 80
HLA-DR	TU36	Q22158	Qdot(112Cd)	Invitrogen	1: 200

*Intracellular staining.

Supplementary Table 5: scRNAseq of differentially expressed genes in LC (n=8) vs. R (n=4) (p < 0.1)

Gene	Cluster	Fold-change	Direction*	Adjusted p-value
THEMIS	1 ($CD8^+$ T cells/CTLs)	1.6	up	0.032
NUDT2	1	1.6	up	0.032
HMGB2	1	1.3	up	0.074
DPM3	1	1.2	up	0.087
NSMAF	1	1.2	up	0.087
ABCD2	1	1.7	up	0.087
TNFRSF18 (GITR)	1	0.5	down	0.074
PHTF1	1	0.7	down	0.074
TSPAN3	1	0.7	down	0.074
MARCH3	1	0.5	down	0.087
STAG3	1	0.4	down	0.087
PPIE	3 (Monocytes)	1.5	up	0.021
NIBAN1	7 ($CD4^+$ T cells)	1.8	up	0.084
CAST	7	1.5	up	0.084
APBA2	7	0.6	down	0.084
RNF157-AS1	7	0.3	down	0.098

*"up" corresponds to upregulated in LC as compared to R, "down" corresponds to down-regulated in LC as compared to R

Supplementary Table 6: Differential GO pathways in Cluster 3 (monocytes) in LC (n=8) vs. R (n=4)

GO pathway	GO ID	Size	Adjusted p-value
mRNA splicing, via spliceosome	GO:0000398	437	0.021
protein peptidyl-prolyl isomerization	GO:0000413	48	0.021
transcription-coupled nucleotide-excision repair	GO:0006283	85	0.021
regulation of transcription, DNA-templated	GO:0006355	535	0.021
protein refolding	GO:0042026	45	0.021
neutrophil degranulation	GO:0043312	482	0.021
positive regulation of viral genome replication	GO:0045070	29	0.021

GO pathway names and ID numbers are listed. Size corresponds to the number of annotated genes in the GO process. Adjusted p-values correspond to the Family-Wise Error Rate (FWER) adjusted p-values.

Supplementary Table 7: Differential GO pathways in Cluster 1 ($CD8^+$ T cells / CTLs) in LC (n=8) vs. R (n=4)

GO pathway	GO ID	Size	Adjusted p-value
nucleobase-containing compound metabolic process	GO:0006139	33	0.057
apoptotic process	GO:0006915	567	0.057
cellular response to oxidative stress	GO:0034599	105	0.057
adaptive immune response	GO:0002250	342	0.064
T cell receptor signaling pathway	GO:0050852	195	0.064

GO pathway names and ID numbers are listed. Size corresponds to the number of annotated genes in the GO process. Adjusted p-values correspond to the Family-Wise Error Rate (FWER) adjusted p-values.

Supplementary Table 8: scRNAseq of differentially expressed genes in *OR7D2*^{high} (n=4) vs. R (n=4) ($p < 0.05$)

Gene	Cluster	Fold-change	Direction*	Adjusted p-value
HIST1H2AM	0 ($CD4^+$ T cells)	2.7	up	0.001
HIST2H2AC	0	1.8	up	0.034
CR1	0	3.4	up	0.034
FGD5-AS1	0	1.4	up	0.034
SVIL	0	1.6	up	0.034
PIP4K2A	0	1.3	up	0.036
NORAD	0	1.4	up	0.036
NUDT2	0	1.6	up	0.038
IFFO2	0	1.5	up	0.039
DENND1B	0	1.3	up	0.039
AC147067.1	0	1.8	up	0.039
RASSF6	0	3.4	up	0.039

ARL15	0	1.3	up	0.039
ARRDC3-AS1	0	1.7	up	0.039
AHNAK	0	1.8	up	0.039
HIST1H2AK	0	2.1	up	0.044
SPCS3	0	1.3	up	0.047
HIST1H2AM	1 (CD8$^+$ T cells/CTLs)	1.6	up	0.004
ITGB1	5 (CD8$^+$ T cells)	1.4	up	0.026
AHNAK	5	8.0	up	0.026
S100A6	5	2.9	up	0.037
HIST1H1E	5	1.8	up	0.037
HIST1H2AM	5	1.7	up	0.037
H1FX	5	1.5	up	0.037
KLF6	5	1.8	up	0.037
MYO1F	5	3.2	up	0.037
TTC39C	5	1.6	up	0.040
IGKV2-24	8 (B cells)	1.7	up	0.011
APOO	0	2.4	down	0.034
MTFP1	0	1.7	down	0.036
AC090360.1	0	1.6	down	0.039
APOO	5	2.1	down	0.026
PECAM1	5	1.6	down	0.037
APOO	7 (CD4$^+$ T cells)	143.1	down	0.027
RGPD2	8 (B cells)	5.1	down	0.002

*"up" corresponds to upregulated in *OR7D2*^{high} LC as compared to R, "down" corresponds to down-regulated in *OR7D2*^{high} LC as compared to R

Supplementary Table 9: scRNAseq of differentially expressed genes in *ALAS2*^{high} (n=4) vs. R (n=4) ($p < 0.05$)

Gene	Cluster	Fold-change	Direction*	Adjusted p-value
NOTCH2NLB	1 (CD8$^+$ T cells/CTLs)	188.1	up	0.007

PDE3A	1	1.7	up	0.012
THEMIS	1	1.7	up	0.033
IGKV3-11	6 (B cells)	6.6	up	0.019
RMRP	7 ($CD4^+$ T cells)	5.5	up	0.042
IGKV2D-40	8 (B cells)	2.0	up	0.025
ME1	1	3.0	down	0.007
SLC4A10	1	33.6	down	0.033
CXXC5	1	2.5	down	0.040
NOG	5 ($CD8^+$ T cells)	1.8	down	0.046
BACH2	7	3.1	down	0.042
NOG	7	469.8	down	0.042
CLECL1	8	3.8	down	0.025
COPDA1	8	5.2	down	0.038

*"up" corresponds to upregulated in *ALAS2*^{high} LC as compared to R, "down" corresponds to down-regulated in *ALAS2*^{high} LC as compared to R

Supplementary Table 10: Differential GO pathways in *OR7D2*^{high} (n=4) vs. R (n=4)

GO pathway	GO ID	Size	Adjusted p-value	Cluster
regulation of RNA splicing	GO:0043484	36	0.030	5
protein complex oligomerization	GO:0051259	71	0.030	5
regulation of voltage-gated calcium channel activity	GO:1901385	8	0.030	5
lipid transport	GO:0006869	63	0.068	5
cristae formation	GO:0042407	34	0.068	5
G1/S transition of mitotic cell cycle	GO:0000082	112	0.077	5
in utero embryonic development	GO:0001701	200	0.077	5
cell fate specification	GO:0001708	37	0.077	5
cell migration involved in sprouting angiogenesis	GO:0002042	22	0.077	5
phagocytosis	GO:0006909	62	0.077	5
cellular defense response	GO:0006968	52	0.077	5
cell adhesion	GO:0007155	472	0.077	5
homophilic cell adhesion via plasma membrane adhesion molecules	GO:0007156	178	0.077	5

leukocyte cell-cell adhesion	GO:0007159	27	0.077	5
cell-matrix adhesion	GO:0007160	105	0.077	5
calcium-independent cell-matrix adhesion	GO:0007161	4	0.077	5
integrin-mediated signaling pathway	GO:0007229	110	0.077	5
positive regulation of cell proliferation	GO:0008284	585	0.077	5
germ cell migration	GO:0008354	9	0.077	5
visual learning	GO:0008542	48	0.077	5
cell migration	GO:0016477	238	0.077	5
cytokine-mediated signaling pathway	GO:0019221	330	0.077	5
formation of radial glial scaffolds	GO:0021943	4	0.077	5
cell projection organization	GO:0030030	37	0.077	5
lamellipodium assembly	GO:0030032	37	0.077	5
B cell differentiation	GO:0030183	76	0.077	5
extracellular matrix organization	GO:0030198	258	0.077	5
positive regulation of cell migration	GO:0030335	259	0.077	5
cell-substrate adhesion	GO:0031589	18	0.077	5
receptor internalization	GO:0031623	45	0.077	5
cell adhesion mediated by integrin	GO:0033627	27	0.077	5
cell-cell adhesion mediated by integrin	GO:0033631	5	0.077	5
heterotypic cell-cell adhesion	GO:0034113	31	0.077	5
negative regulation of Rho protein signal transduction	GO:0035024	27	0.077	5
positive regulation of apoptotic process	GO:0043065	383	0.077	5
positive regulation of GTPase activity	GO:0043547	342	0.077	5
sarcomere organization	GO:0045214	49	0.077	5
negative regulation of cell differentiation	GO:0045596	47	0.077	5
positive regulation of angiogenesis	GO:0045766	173	0.077	5

viral entry into host cell	GO:0046718	87	0.077	5
mesodermal cell differentiation	GO:0048333	12	0.077	5
axon extension	GO:0048675	26	0.077	5
dendrite morphogenesis	GO:0048813	38	0.077	5
regulation of immune response	GO:0050776	213	0.077	5
modulation of chemical synaptic transmission	GO:0050804	62	0.077	5
leukocyte migration	GO:0050900	191	0.077	5
leukocyte tethering or rolling	GO:0050901	21	0.077	5
regulation of cell cycle	GO:0051726	121	0.077	5
positive regulation of protein kinase B signaling	GO:0051897	191	0.077	5
cardiac muscle cell differentiation	GO:0055007	22	0.077	5
cellular response to low-density lipoprotein particle stimulus	GO:0071404	20	0.077	5
basement membrane organization	GO:0071711	11	0.077	5
positive regulation of protein localization to plasma membrane	GO:1903078	55	0.077	5
positive regulation of signaling receptor activity	GO:2000273	6	0.077	5
negative regulation of anoikis	GO:2000811	18	0.077	5
lipid transport	GO:0006869	63	0.027	7
cristae formation	GO:0042407	34	0.027	7
NLS-bearing protein import into nucleus	GO:0006607	19	0.002	8
positive regulation of GTPase activity	GO:0043547	342	0.002	8
adaptive immune response	GO:0002250	342	0.022	8
immunoglobulin production	GO:0002377	91	0.022	8
immune response	GO:0006955	461	0.022	8

GO pathway names and ID numbers are listed. Size corresponds to the number of annotated genes in the GO process. Adjusted p-values correspond to the Family-Wise Error Rate (FWER) adjusted p-values. The last column lists the cell cluster the analysis originated from.

Supplementary Table 11: Differential GO pathways in *ALAS2*^{high} (n=4) vs. R (n=4)

GO pathway	GO ID	Size	Adjusted p-value	Cluster
nucleobase-containing compound metabolic process	GO:0006139	33	0.057	1
apoptotic process	GO:0006915	567	0.057	1
cellular response to oxidative stress	GO:0034599	105	0.057	1
adaptive immune response	GO:0002250	342	0.064	1
T cell receptor signaling pathway	GO:0050852	195	0.064	1

GO pathway names and ID numbers are listed. Size corresponds to the number of annotated genes in the GO process. Adjusted p-values correspond to the Family-Wise Error Rate (FWER) adjusted p-values. The last column lists the cell cluster the analysis originated from.

Supplementary Table 12: T-scan peptides used in study

Peptide	Sequence	Parent protein	Molecular Weight
1	KLWAQCVQL	ORF 1ab	1088.33
2	YLQPRTFLL	S	1150.39
3	LLYDANYFL	ORF 3a	1131.29
4	ALWEIQQVV	ORF 1ab	1085.27
5	LLLDRLNQL	N	1097.32
6	YLFDESGEFKL	ORF 1ab	1347.49
7	FTSDYYQLY	ORF 3a	1199.28
8	TTDPSFLGRY	ORF 1ab	1156.26
9	PTDNYITTY	ORF 1ab	1087.15
10	ATSRTLSYY	M	1061.16
11	NTCDGTTFTY	ORF 1ab	1122.17
12	DTDFVNEFY	ORF 1ab	1149.18
13	GTDLEGNFY	ORF 1ab	1015.04
14	KTFPPTEPK	N	1044.22
15	KCYGVSPTK	S	982.16
16	MVTNNTFTLK	ORF 1ab	1168
17	KTIQPRVEK	ORF 1ab	1098.31
18	KTFPPTEPK	N	1044.22
19	VTDTPKGPK	ORF 1ab	942.08

20	ATEGALNTPK	N	1001.1
21	ASAFFGMSR	N	973.11
22	ATSRTLSYYK	M	1189.33
23	QYIKWPWYI	S	1296.53
24	VYFLQSINF	ORF 3a	1130.31
25	VYIGDPAQL	ORF 1ab	975.11
26	SPRWYFYYL	N	1294.47
27	RPDTRYVL	ORF 1ab	1019.17
28	IPRRNVATL	ORF 1ab	1039.25

N: Nucleoprotein.
M: Membrane protein.
S: Spike protein.
ORF: Open reading frames.

Supplementary Table 13: Flow cytometry antibodies used in study

Antibody	Clone	Catalog	Fluorochrome	Manufacturer	Dilution
CD3	UCHT1	11-0038-41	FITC	Invitrogen	1: 200
CD4	SK3	566356	BV750	BD Biosciences	1: 200
CXCR4	12G5	17-9999-41	APC	Invitrogen	1: 200
CXCR5	J252D4	356919	BV421	BioLegend	1: 200
CCR6	11A9	562724	BV605	BD Biosciences	1: 200
CD45RO	UCHL1	304227	APC Cy7	BioLegend	1: 200
live/dead	NA	423107	zombie UV	BioLegend	1: 500
CD3	UVHT1	612941	BUV496	BD Biosciences	1: 200
CD8a	RPA-T8	301005	FITC	BioLegend	1: 150
PD-1	EH12-2H7	329965	BV750	BioLegend	1: 200
CTLA4*	L3D10	349913	PE-Cy7	BioLegend	1: 200
IFNγ*	B27	566395	BB700	BD Biosciences	1: 200
TNF*	Mab11	502937	BV650	BioLegend	1: 200
CCL4*	D21-1351	562900	BV421	BD Biosciences	1: 200
live/dead	NA	423105	zombie NIR	BioLegend	1: 500
CD28	L293	340975	NA	BD Biosciences	1: 500
CD49d	L25	340976	NA	BD Biosciences	1: 1000

*Intracellular staining.
