# Supplementary material for: Long COVID manifests with T cell dysregulation, inflammation and an uncoordinated adaptive immune response to SARS-CoV-2
Source: Nat Immunol. 2024 Jan 11;25(2):218–25. doi: 10.1038/s41590-023-01724-6 (PMC10834368; doi:10.1038/s41590-023-01724-6)
Supplement: Supplementary file 2 — Reporting Summary [file 41590_2023_1724_MOESM2_ESM.pdf]

nature portfolio

Corresponding author(s): Nadia R. Roan

Last updated by author(s): Nov 20, 2023

Reporting Summary

Nature Portfolio wishes to improve the reproducibility of the work that we publish. This form provides structure for consistency and transparency in reporting. For further information on Nature Portfolio policies, see our Editorial Policies and the Editorial Policy Checklist.

Statistics

For all statistical analyses, confirm that the following items are present in the figure legend, table legend, main text, or Methods section.

n/a	Confirmed	
☐	☒	The exact sample size (*n*) for each experimental group/condition, given as a discrete number and unit of measurement
☐	☒	A statement on whether measurements were taken from distinct samples or whether the same sample was measured repeatedly
☐	☒	The statistical test(s) used AND whether they are one- or two-sided<br>*Only common tests should be described solely by name; describe more complex techniques in the Methods section.*
☒	☐	A description of all covariates tested
☐	☒	A description of any assumptions or corrections, such as tests of normality and adjustment for multiple comparisons
☐	☒	A full description of the statistical parameters including central tendency (e.g. means) or other basic estimates (e.g. regression coefficient) AND variation (e.g. standard deviation) or associated estimates of uncertainty (e.g. confidence intervals)
☐	☒	For null hypothesis testing, the test statistic (e.g. *F*, *t*, *r*) with confidence intervals, effect sizes, degrees of freedom and *P* value noted<br>*Give P values as exact values whenever suitable.*
☒	☐	For Bayesian analysis, information on the choice of priors and Markov chain Monte Carlo settings
☐	☒	For hierarchical and complex designs, identification of the appropriate level for tests and full reporting of outcomes
☐	☒	Estimates of effect sizes (e.g. Cohen's *d*, Pearson's *r*), indicating how they were calculated

Our web collection on statistics for biologists contains articles on many of the points above.

Software and code

Policy information about availability of computer code

Data collection	No software was used for data collection.
Data analysis	The Seurat package version 4.3.0 was used for scRNAseq analysis. FlowJo (version 10.8.1) was used for CyTOF data analyses.

For manuscripts utilizing custom algorithms or software that are central to the research but not yet described in published literature, software must be made available to editors and reviewers. We strongly encourage code deposition in a community repository (e.g. GitHub). See the Nature Portfolio guidelines for submitting code & software for further information.

Data

Policy information about availability of data

All manuscripts must include a data availability statement. This statement should provide the following information, where applicable:

- Accession codes, unique identifiers, or web links for publicly available datasets
- A description of any restrictions on data availability
- For clinical datasets or third party data, please ensure that the statement adheres to our policy

The raw CyTOF datasets for this study corresponding to total and SARS-CoV-2-specific CD4+ and CD8+ T cells, as well as the raw Olink data, are publicly accessible through the following link: https://datadryad.org/stash/share/TE_QuY0JX23V2n2CIMO2PgsR6afIp6GGusdQ5nXVGnk. The human reference genome (GRCh38) was used for alignment. The raw bulk RNAseq and scRNAseq data from this study are deposited in the GEO (Gene Expression Omnibus) database: GSE224615 (for bulk RNAseq) and GSE235050 (for scRNAseq).

Human research participants

Policy information about studies involving human research participants and Sex and Gender in Research.

Reporting on sex and gender	Sex as a biological variable were considered in this study. Data were analyzed by biological sex at birth, as determined by self-reporting. Sex-disaggregated data are presented in the manuscript.
Population characteristics	Our research home is at San Francisco General Hospital, a large, public safety-net hospital that serves individuals with limited access to healthcare services. Our research center is located in the heart of the Mission, a working-class Latino community, and adjacent to Bayview, a working-class Black community. All the investigators are committed to promoting inclusivity and equity. The SARS-CoV-2 pandemic in San Francisco has disproportionately affected communities of color. Our team has a long history of engaging affected communities in research. Our clinical research home at San Francisco General Hospital (SFGH) has a social justice mission and serves as the safety net public hospital for the Bay Area. LIINC is highly diverse including participants who identify as cisgender women (45%), transgender men and women (3% and 2%, respectively), Latino (38%) including monolingual Spanish speakers (14%), Black (5%), Asian (15%), Pacific Islander (5%) and Native American (3%). The median age of our participants was 46 (46 among LC group, 45.5 among non-LC group). A subset of our participants experience physical disabilities, as well as limitations related to ME/CFS, dysautonomia, and post-exertional malaise. Our team is highly diverse and includes individuals from a variety of backgrounds including immigrants to the U.S., people of color, and LGBTQ+ individuals. We have a diverse and inclusive Community Advisory Board, representing the full spectrum of community, sociodemographic, and sexual identity. We believe that our Long COVID work gives agency and voice to individuals experiencing this medically unexplained condition. All recruitment materials, our study website, and study instruments are available in both English and Spanish, and we are currently developing Spanish-language materials to disseminate preliminary study findings to participants. Retention in the cohort is high; over 85% of participants remain in the study after the first 14 months.
Recruitment	Details of cohort recruitment, enrollment, and measurement procedures have been described in detail previously (PMID 35106317). Briefly, LIINC is a prospective observational study enrolling individuals with prior nucleic acid-confirmed SARS-CoV-2 infection in the San Francisco Bay Area, regardless of the presence or absence of post-acute symptoms. At each study visit, participants underwent an interviewer-administered assessment of 32 physical symptoms that were newly developed or had worsened since COVID-19 diagnosis, as well as assessment of mental health and quality of life. Pre-existing and unchanged symptoms were not considered to be attributable to COVID-19. In addition, detailed data regarding medical history, COVID-19 history, SARS-CoV-2 vaccination, and SARS-CoV-2 reinfection were collected. Two participants enrolled in LIINC had biospecimens collected previously via the UCSF COVID-19 Host Immune Response Pathogenesis (CHIRP) study, which utilizes identical procedures for ascertainment of clinical history as the LIINC study (PMID 34636722).
Ethics oversight	The study protocol was approved by the UCSF Institutional Review Board (IRB).

Note that full information on the approval of the study protocol must also be provided in the manuscript.

Field-specific reporting

Please select the one below that is the best fit for your research. If you are not sure, read the appropriate sections before making your selection.

☒ Life sciences ☐ Behavioural & social sciences ☐ Ecological, evolutionary & environmental sciences

For a reference copy of the document with all sections, see nature.com/documents/nr-reporting-summary-flat.pdf

Life sciences study design

All studies must disclose on these points even when the disclosure is negative.

Sample size	No sample-size calculation was performed, as this is the first study to perform this kind of analysis, and well-annotated specimens from well-characterized and individuals with a clear post-acute sequelae of SARS-CoV-2 infection diagnosis are limited.
Data exclusions	No data were excluded from the analyses.
Replication	Reproducibility of experimental findings was established as detailed in the manuscript. For each assay, each donor was only measured once; hence all replicates corresponded to biological replicates, and not technical replicates. These biological replicates ranged from n=4 to n=27, as detailed within the manuscript. Of note, only statistically significant findings were used to draw conclusions.
Randomization	This is not pertinent to our study, because our study was not a clinical trial. In addition, we could not randomize since we were comparing two patient groups: LC vs. non-LC.
Blinding	Blinding was not appropriate for our study, as the CyTOF data needed to be generated in multiple batches, and equal distribution of study groups between batches was established so as to minimize the effect of batch on data outcome. This required knowing which samples belonged to which patient group. The RNAseq and scRNAseq data were run in single batches each.

Reporting for specific materials, systems and methods

We require information from authors about some types of materials, experimental systems and methods used in many studies. Here, indicate whether each material, system or method listed is relevant to your study. If you are not sure if a list item applies to your research, read the appropriate section before selecting a response.

Materials & experimental systems

n/a	Involved in the study
☐	☒ Antibodies
☒	☐ Eukaryotic cell lines
☒	☐ Palaeontology and archaeology
☒	☐ Animals and other organisms
☒	☐ Clinical data
☒	☐ Dual use research of concern

Methods

n/a	Involved in the study
☒	☐ ChIP-seq
☐	☒ Flow cytometry
☒	☐ MRI-based neuroimaging

Antibodies

Antibodies used	Detailed information about antibodies used, including antibody dilutions and vendors are provided in the Tables and Methods sections.
Validation	All antibodies were validated by CyTOF on multiple cell types, prior to their application on the test samples. In particular, antibodies were tested on single-cell suspensions generated from fresh human tonsils, where expression of each antigen was compared between B cells and T cells, or different subset of T cells. Only batches of antibodies staining as expected based on known expression on cellular subsets were used. The validation studies are depicted in Extended Figure 10.

Flow Cytometry

Plots

Confirm that:

☒ The axis labels state the marker and fluorochrome used (e.g. CD4-FITC).

☒ The axis scales are clearly visible. Include numbers along axes only for bottom left plot of group (a 'group' is an analysis of identical markers).

☒ All plots are contour plots with outliers or pseudocolor plots.

☒ A numerical value for number of cells or percentage (with statistics) is provided.

Methodology

Sample preparation	*Describe the sample preparation, detailing the biological source of the cells and any tissue processing steps used.*
Instrument	*Identify the instrument used for data collection, specifying make and model number.*
Software	*Describe the software used to collect and analyze the flow cytometry data. For custom code that has been deposited into a community repository, provide accession details.*
Cell population abundance	*Describe the abundance of the relevant cell populations within post-sort fractions, providing details on the purity of the samples and how it was determined.*
Gating strategy	*Describe the gating strategy used for all relevant experiments, specifying the preliminary FSC/SSC gates of the starting cell population, indicating where boundaries between "positive" and "negative" staining cell populations are defined.*

☐ Tick this box to confirm that a figure exemplifying the gating strategy is provided in the Supplementary Information.

March 2021
